# Cannabis, psychosis, and violence: case reports and comprehensive evidence review

**DOI:** 10.3389/fpsyt.2026.1824748

**Published:** 2026-07-21

**Authors:** Kenneth P. Finn, Robin M. Murray, Elizabeth Stuyt, Karen Randall

**Affiliations:** 1 School of Medicine, Creighton University, Omaha, NE, United States; 2 NIHR Maudsley Biomedical Research Centre, London, United Kingdom; 3 International Academy on the Science and Impacts of Cannabis, St. Petersburg, FL, United States

**Keywords:** cannabis, cannabis-induced psychosis, harm, psychosis, violence

## Abstract

Cannabis is one of the most widely used psychoactive substances, with higher use rates in countries that have implemented policies to legalize medical or recreational use. Following the development of cannabis products with concentrations well above 10% THC (high potency, delta-9 tetrahydrocannabinol), the number of psychosis cases has also increased. Cannabis-attributable psychosis cases increased from 1995 to 2010, and speculates that increased cannabis potency might have contributed. Psychosis is a complex disorder regarding its etiology, with multiple factors well documented and accepted, including genetic predisposition, adverse childhood events, and recreational drug use. Cannabis is a potential contributing factor to psychosis or subsequent violence, but toxicology in such cases often does not include THC. Here we provide a perspective to argue that greater attention should be put toward cannabis-associated violence. We present several high-profile cases of violence reported in main-stream media, where the only substance involved is tetrahydrocannabinol (THC) or the involvement of cannabis was noted in the person’s history. Some of these cases have well-documented cannabis use preceding the onset of psychosis and violence, yet toxicology was not done or did not include THC. The scientific evidence regarding the relationship between cannabis, psychosis, and violence continues to evolve and should be part of medical screening, law-enforcement concerns, and policymaking.

## Case reports

Recent research suggests that the number of psychosis cases associated with cannabis use has increased in the last two decades (1, 2). The increase coincides with an increase in concentrations of delta-9 tetrahydrocannabinol in cannabis well above 10% THC (3, 4). Here we report cases of cannabis-associated violence that were first identified by reports in the media. Authors generated a list of violence cases, and further investigation was done using internet searches of media reports to determine if cannabis use was thought to be involved and if the perpetrators showed symptoms of psychosis. Attempts were made through the Freedom of Information Act to obtain toxicology results. Early cases were mostly located in Colorado, the first US state to legalize retail cannabis. However, now such cases are reported more broadly across the US. There is some commonality in many of the violence cases. Family members and friends often reported that there was a change in the perpetrator’s behavior and thinking over time, including paranoid ideations, delusions, or auditory hallucinations. Often the behavior includes relieving themselves of clothing and appearing naked. Common themes involve use of guns, edged weapons, severe interpersonal violence, or other homicidal behavior. The violent act is frequently unanticipated, with individuals acquainted with the perpetrator expressing disbelief that he or she could have engaged in such behavior.

**Table 1** includes media-reported cases of significant violence where THC is the only drug present in toxicology or there is a diagnosis of psychosis. **Table 2** includes media-reported cases of violent behavior where testing for THC was not done or not reported, despite evidence that the perpetrator had been using cannabis.

**Table 1:** Cases of Significant Unexpected Violence Where THC Was the Only Drug in Toxicology.

Year	Situation	Event/Crime	Toxicology
2012	18 yr old male	Death by Suicide	(+) THC
2012	26 yr old male	Accidental Death	(+) THC
2014	19 yr old male	Accidental Death	(+) THC
2014	47 yr old male	Second Degree Murder	(+) THC
2015	33 yr old male	Multiple homicides	(+) THC
2016	39 yr old male	Homicide e the home.	(+) THC
2017	44 yr old male	Multiple homicides	(+) THC
2018	32 yr old female	Homicide	(+) THC
2018	31 yr old male	Homicide psychosis	He admitted he was a heavy cannabis-user.
2018	21 yr old male	Homicide	(+) THC
2018	28 yr old male	Multiple homicides	(+) THC
2019	45 yr old male	Multiple homicides	(+) THC
2020	44 yr old male	Homicide	(+) THC
2021	35 yr old male	Homicide	Cannabis-induced psychosis
2021	20 yr old male	Suicide	(+) THC
2021	19 yr old male	Multiple homicides	(+) THC
2021	30 yr old male	Homicide	(+) THC

**Table 2:** Violent Event Where Testing for THC Was Not Done or Not Reported – underscores need for better testing, even mandates.

Date	Situation	Event/Crime	Comments on Toxicology
2017	45 yr old male	Multiple homicides	Past convictions for marijuana possession
2018	33 yr old male	Multiple homicides	Planned to drive to a marijuana state, describing a life in which he would smoke marijuana
2018	19 yr old male	Multiple homicides	Admitted using “a lot” of marijuana
2019	24 yr old male	Multiple homicides	Was addicted to marijuana since high school.
2019	44 yr old male	Multiple homicides	Seen smoking marijuana
2019	21 yr old male	Multiple homicides	Had a history of smoking marijuana for years
2020	51 yr old male	Multiple homicides	Toxicology acetaminophen and caffeine. THC not included in toxicology.
2020	31 yr old male	Multiple homicides	Told others that he had recently run out of marijuana shortly before the shooting
2020	24 yr old male	Homicide	Two psychiatric evaluations concluded cannabis use disorder
2021	25 yr old male	Drowning	(+) THC
2021	47 yr old male	Multiple homicides	Had a marijuana growing operation

## Discussion

### Cannabis and psychosis

The evidence consistently demonstrates that cannabis use, particularly use of high-potency products, and particularly if started during adolescence, is associated with increased risk of psychotic disorders. Cannabis-induced psychosis has the highest conversion rate to schizophrenia (34-47%) among all substance-induced psychoses (5–7). Adolescents ages 12–19 who use cannabis have an 11-fold increased risk of psychotic disorders, rising to 26.7-fold for severe outcomes requiring hospitalization (8). Following cannabis legalization in Canada, emergency department visits for cannabis-induced psychosis increased by 40.4%, with the highest rates among young adults ages 19-24 (9). Daily use of high-potency cannabis exceeding 10% THC is associated with 5-fold higher risk of psychotic disorder compared with no use (10).

### Neurobiological mechanisms

THC acts as a partial agonist at CB1 receptors, which are highly concentrated in the prefrontal cortex, hippocampus, and basal ganglia. During adolescence, the endocannabinoid system plays critical roles in synaptic pruning, myelination, and neurotransmitter regulation. Exogenous cannabinoid exposure during this period may disrupt these developmental processes, potentially leading to alterations in brain structure and function (11, 12). Cannabis use during adolescence has been hypothesized to influence neurodevelopment, including potential effects on dendritic spine pruning in prefrontal cortex pyramidal neurons and on the balance of excitatory and inhibitory neurotransmission. These changes may contribute to alterations in executive function, memory consolidation, and sensory gating--processes that are also impaired in schizophrenia.

Cannabis use during adolescence may cause early pruning of dendritic spines in prefrontal cortex pyramidal neurons and disrupt the balance of excitatory and inhibitory neurotransmission, affecting executive function, memory consolidation, sensory gating, and other cognitive processes impaired in schizophrenia (13, 14). This evidence is largely based on animal models, with no or limited evidence in humans.

Mechanisms linking cannabis use to violence include acute psychotic reactions with paranoid delusions (15), impaired impulse control and neurobiological changes affecting dopaminergic systems and synaptic density (16, 17). Recent neuroimaging research has revealed that cannabis use disorder is associated with elevated dopamine function in brain regions implicated in psychosis. A 2025 study using neuromelanin-sensitive MRI which is a proxy measure of dopamine levels, found that individuals with cannabis use disorder showed elevated neuromelanin-MRI signal in ventral substantia nigra and ventral tegmental area voxels, with a dose-dependent relationship where by higher symptom burden correlated with higher neuromelanin signal (18). This finding suggests that cannabis use may affect the same dopaminergic pathway that is disrupted in schizophrenia, providing a mechanistic link between cannabis exposure and psychotic symptoms. Biological plausibility is also supported by cannabis administration studies showing THC causes transient psychotic-like experiences.

An important clinical finding is that psychosis can occur not only during cannabis intoxication but also during withdrawal. A 2025 systematic review identified 112 cases of withdrawal-associated psychosis, typically occurring within one week of cessation with peak risk at 4 days in daily cannabis users. Sleep difficulty was present in 90% of cases. Individuals who resumed cannabis use had 13.9 times higher relapse risk than those who maintained abstinence, suggesting gradual tapering rather than abrupt cessation may be safer (19).

### Cannabis-induced psychosis and conversion to schizophrenia

Multiple systematic reviews have established that cannabis-induced psychosis has the highest rate of conversion to schizophrenia compared to other substance-induced psychoses. A 2019 meta-analysis examining 50 studies with 40,783 individuals found that 34% of cannabis-induced psychosis cases transitioned to schizophrenia, significantly higher than alcohol (10%), sedatives (9%), or opioids (12%) (20). Danish registry data corroborated these findings, showing 47.4% of cannabis-induced psychosis patients converted to either schizophrenia or bipolar disorder within the study period, with half of schizophrenia conversions occurring within 3.1 years of the initial substance-induced episode (6).

A population-based Ontario study of 9.8 million individuals found that people who visited emergency department for cannabis-induced psychosis were 84 times more likely to be subsequently diagnosed with schizophrenia compared to the general population. Among males aged 14–18 with cannabis-induced psychosis, over 40% developed schizophrenia within the follow-up period (21).

### Age-dependent vulnerability

The most striking finding in recent research is the profound age-dependent nature of cannabis-related psychosis risk. A groundbreaking 2024 Canadian study of 11,363 youth found that cannabis use during adolescence (ages 12-19) was associated with an 11.2-fold increased risk of psychotic disorders. When restricted to severe outcomes requiring hospitalization or emergency department visits, this risk increased dramatically to 26.7-fold (8). Remarkably, this association was not apparent in young adults (ages 20-33), for whom no significant relationship was found. This suggests a critical neurodevelopmental window during adolescence when the brain is particularly susceptible to cannabis-related harm. The study found that 77.8% of adolescent psychotic disorder hospitalizations occurred in individuals with past-year cannabis use.

Modern cannabis, which may contain potencies reaching 70-90% THC, potentially poses substantially greater psychiatric risks than historical products containing less than 4% THC (22, 23). A systematic review analyzing adolescent cannabis use found that both high-frequency and low-frequency use during ages 12–18 were associated with significantly increased odds of schizophrenia (24). Cannabis use at age 15 or younger showed particularly strong associations with psychotic disorder development, consistent with neurotoxic effects during critical brain maturation periods, although there are alternative explanations to neurotoxic effects, such as common genetic liability. Further, adolescent use is a severity marker – part of a broader early-risk phenotype. It is also important to understand that violence in people under the age of 18 is uncommon, however, the concern is whether early-onset use and its associated psychiatric effects may have an impact on violence later into young adulthood.

### Cannabis potency and psychiatric risk

THC content in cannabis has increased dramatically over recent decades. From the 1960s to the 1980s, cannabis typically contained 2-4% THC. Currently, cannabis flower in some regions of the United States averages 20% THC (25), with concentrates reaching 80-90% THC (26). In Canada’s legal market, 71% of dried cannabis sold in 2022 contained THC levels exceeding 20% (27). A 2025 systematic review of 99 studies involving 221,097 participants found that high-concentration THC products showed consistent unfavorable associations with psychosis and schizophrenia (28). Daily users of high-potency cannabis exceeding 10% THC have approximately 5 times higher risk of psychotic disorder compared with never-users, with a clear dose-response relationship between potency and psychiatric outcomes (29).

### Post-legalization trends

Following recreational cannabis legalization in Canada in October 2018, emergency department visits for cannabis-induced psychosis increased approximately 40% during the commercialization period. The study tracked data from 2010–2022 and found the most pronounced increases among young adults aged 19–24 years (30).

Recreational cannabis law implementation in Massachusetts was associated with an increase in cannabis use after retail outlets opened (31). These data suggest that increased availability and commercialization contribute to higher rates of cannabis-related psychiatric emergencies.

A Denver Health study examining psychosis hospitalizations from 2005–2020 found significant increases following policy changes (32). Psychosis hospitalization rates among youth increased from 21.9 per 100,000 pre-legalization to 32.3 per 100,000 post-legalization. For psychosis hospitalizations involving cannabis use disorder specifically, rates increased from 2.0 per 100,000 to 7.9 per 100,000.

Although violence is rare among people who use cannabis and among people diagnosed with schizophrenia, persistent cannabis use is an independent risk factor for violent behaviors in patients with schizophrenia. Investigators followed 965 patients longitudinally and found that persistent cannabis use predicted subsequent violence, but violence did not predict cannabis use. The relationship was unidirectional and persisted when controlling for stimulants and alcohol use (33).

### Cannabis and violence

The current evidence on the relationship between cannabis use and violent behavior has been documented through comprehensive examination of epidemiological studies, meta-analyses, neurobiological research, and clinical case series. There are data to suggest that cannabis or cannabinoid use may reduce the likelihood of violence, whereas other data may suggest the contrary, underscoring the complex nature of the relationship between cannabis use and violence, particularly discontinuation of chronic use (34). The complexity of this has been demonstrated in animal models, suggesting mixed effect on aggression, and may dependent upon chronicity of use, dose, and environmental factors (35).

In 2002, Raphael Mechoulam, the researcher credited with the discovery of the cannabinoid receptors and the subsequent discovery of the endocannabinoid system, reported on the unexpected production of aggression by cannabis and cannabinoids under stressful conditions. Preclinical research demonstrated that cannabis extract given to rats already stressed by cold or by abstinence from previously administered morphine induced irritability or aggressiveness. Cannabis also caused aggression in rats deprived of REM sleep for 4 days. Chronic THC or even a single THC injection could induce mouse killing behavior in previously ‘non-killer’ rats (36, 37).

In meta-analyses, cannabis has been associated with violence, yet the exact mechanisms have still not been completed elucidated. The association has also been demonstrated in intimate partner violence and pre- and post- legalization trends should be further investigated, and future studies should include at-risk populations, such as those with serious mental illness, as well as biological studies, such as neuroimaging (38). Cannabis use is associated with aggression or violence in people with PTSD and or psychotic-spectrum disorders (39). Studies have shown cannabis use is common in those in the early phase of psychosis and is a risk factor for violent behavior in that population as well as those with schizophrenia. The link between cannabis use and violent behavior may be the impulsivity, which is a risk factor for violent behavior in psychosis. There may be neurobiological mechanisms in play, particularly in areas of the brain modulating impulsivity, such as the frontal areas (40). There may be bidirectional relationships between cannabis use and aggression. There may be underlying biological mechanisms, including environmental and genetic risk factors as well as self-medicating strategies mitigating the emotions associated with reactive aggression (41). This highlights the complex nature of cannabis use and violence.

Other research demonstrates associations between cannabis use and violent behavior in psychiatric populations. A longitudinal study of 1,136 recently discharged psychiatric patients found that persistent cannabis use across multiple follow-up periods predicted violence. Patients who used cannabis at all four follow-up assessments were 2.44 times more likely to display violence than non-users, which was higher than alcohol or cocaine (42). A dose-response relationship has been established: occasional users showed 3% violence rates, regular users showed 6% rates, and frequent users showed 11% violence rates. Each increase in cannabis use frequency profile was associated with 1.91 times higher odds for violence (43).

A study of 3,028 Iraq and Afghanistan-era veterans found that current cannabis use disorder was significantly associated with difficulty managing anger (OR = 2.93), aggressive impulses (OR = 2.74), and problems controlling violence (OR = 2.71). Among veterans with current cannabis use disorder, 27% reported difficulty controlling violence in the past 30 days, compared to only 8% of those without cannabis use disorder (44).

### Clinical implications

While the current evidence on the correlation between cannabis use and violent behavior has been well documented through epidemiological studies, meta-analyses, neurobiological research, and clinical case series, it cannot yet be determined to be causal, particularly in a chronic user. There are cases where the temporal relationship between use and onset of violence is immediate, suggesting that cannabis has a role in its development. We clearly need more research and toxicological data from cases of unintended violence to help determine causality. Research suggests that asking why someone started using cannabis could serve as a simple screening tool for identifying high-risk individuals. Those who began cannabis use for self-medication of anxiety, depression, or physical discomfort showed significantly higher THC consumption and more severe paranoia and mood symptoms compared to those who started for social reasons (45). Prevention efforts should specifically target adolescents, given their dramatically elevated vulnerability. Public health campaigns should emphasize age-specific risks of cannabis use, dangers of high-potency products, association between cannabis and psychotic disorders, and early warning signs of cannabis-induced psychosis.

The primary treatment for cannabis-induced psychosis is abstinence from cannabis, often combined with second-generation antipsychotics. A Thai study of 317 cannabis-induced psychosis patients found that 98.7% received antipsychotics, with significant symptom improvement by day 8 and continued improvement through day 28 (46). Behavioral interventions including motivational interviewing and cognitive behavioral therapy can support cannabis cessation and reduce relapse risk.

There are very few places in the world with a comprehensive approach to the treatment with people with cannabis induced psychosis. The most renowned clinic is the Cannabis Clinic for Patients with Psychosis led by Dr. Marta DiForti, Consultant Psychiatrist from South London and Maudsley NHS Foundation Trust (SLaM) and King’s College London (KCL). With more awareness of this condition, there will hopefully be more clinics worldwide that can model treatment approach like hers.

### Epidemiological findings

Epidemiological studies demonstrate consistent, dose-dependent associations between cannabis use and violent behavior. Young adults with daily cannabis use show 1.7-1.8 times higher rates of violence in males and 1.6-2.4 times higher in females (47). Persistent cannabis use across the lifespan is associated with 7-fold increased odds of violent convictions (48). Cannabis-induced psychosis represents a particularly high-risk state, with 77% violence rates when combined with impulsivity and 53-58% when combined with lack of insight and medication non-adherence (49). Substance abuse, including cannabis, appears to be the primary mediating factor for violence in psychiatric populations (50).

## Limitations

It is difficult infer real-world increases or causal associations from media cases because they are not representative of all events. Media coverage is selective, tends to highlight extreme or newsworthy incidents, and changes over time, creating the appearance of trends that may not reflect actual incidence. In addition, there are no denominators, inconsistent measurement, and uncontrolled confounding, so these cases can generate hypotheses but cannot support conclusions about rates, risk, or causality. There was no systematic review of news media outlets to obtain the case studies. The cases were investigated one at a time and as they arose from mainstream media either by mentions of cannabis use or after pursuing legal avenues to obtain toxicology reports.

A small number of the cases which mentioned cannabis use included toxicology results, but the majority did not. Interestingly, of the cases not mentioning cannabis use and toxicology was able to be obtained, almost all of them did show THC on the toxicology report and rarely other substances, including alcohol, kratom, or other illicit substances. The lack of consistency across the country likely accounts for a significant under reporting of cannabis related violent deaths. For example, based on communication by the authors, the Los Angeles County Coroners Office, an administrative branch to the second largest city in the United States, will not test for cannabinoids as it is not required or part of their screens for violent crime related deaths and is not of concern for cause or manner of death determinations in violent crime related deaths. The state of Maryland will not test for cannabinoids unless associated with an impaired driving fatality and the state of Rhode Island will protect any toxicology report on a perpetrator for 50 years under HIPPA. Other states, like Connecticut, Minnesota, New York, and Nevada will only allow test results be released to next of kin or mandated by the court.

## Conclusions and public health implications

The evidence documenting cannabis-related psychiatric harm has strengthened considerably in recent years. It is important to acknowledge that not everyone who uses cannabis will become violent or psychotic and there is evidence that use may decrease violence, making the relationship even more difficult to determine. The current literature is showing potential increasing cannabis-related risks of psychosis and violence through several possible mechanisms. Cannabis-induced psychosis carries the highest risk of conversion to chronic schizophrenia among all substance-induced psychoses. The relationship is particularly pronounced during adolescence, when neurodevelopmental vulnerability creates dramatically elevated risk. Modern high-potency cannabis products pose greater psychiatric risks than historical cannabis preparations. Post-legalization data from both Canada and Colorado demonstrate measurable increases in cannabis-related psychiatric emergencies, particularly among young adults. The association between cannabis use and violence in psychiatric populations is well-established, with dose-response relationships and persistence effects.

From a clinical perspective, cannabis should be considered a significant modifiable risk factor for psychotic disorders as well as for violent behavior. Healthcare providers should screen for cannabis use in all patients, particularly adolescents and young adults, as well as those with mental health disorders and provide evidence-based education about psychiatric risks. Those who experience cannabis-induced psychosis require long-term follow-up given the high conversion rate to schizophrenia. Future research priorities include studies examining specific THC/CBD ratios and their psychiatric effects, identification of genetic and environmental risk factors for cannabis-induced psychosis, development of targeted prevention strategies for high-risk populations, and evaluation of treatment approaches for cannabis use disorder in patients with psychotic disorders. The convergence of evidence from epidemiological, neurobiological, and clinical studies supports a causal relationship between cannabis use and psychotic disorders and violence, particularly when use begins during adolescence and involves high-potency products. It is important to understand that observational studies are correlational and cannot determine if cannabis causes violence, if people prone to violence selectively use cannabis, or if some third variable causes both cannabis use and violence.

Cannabis use demonstrates potential associations with violence, particularly in individuals with psychiatric disorders, heavy or persistent use patterns, cannabis use disorder, exposure to high-potency (greater than 10% THC) products, and early age of initiation via neurobiological mechanisms which are only now beginning to be understood. The increasing potency of modern cannabis products and expansion of legalization raise significant public health concerns. Evidence supports cannabis as a modifiable risk factor for both psychosis and violence, warranting targeted prevention efforts, particularly for vulnerable populations including adolescents, individuals with psychiatric disorders, and those with family history of psychotic illness. The complex nature of the relationship between cannabis use, mental health effects, including psychosis and its correlation to violence, underscores the need for comprehensive treatment approaches. A mandate to include THC in the toxicology of any unintended episode of violence would help in providing a better understanding of this correlation.

## Data Availability

The original contributions presented in the study are included in the article/supplementary material. Further inquiries can be directed to the corresponding authors.
